# Unlocking the genomic potential of aerobes and phototrophs for the production of nutritious and palatable microbial food without arable land or fossil fuels

**DOI:** 10.1111/1751-7915.13747

**Published:** 2021-02-02

**Authors:** Abbas Alloul, Janne Spanoghe, Daniel Machado, Siegfried E. Vlaeminck

**Affiliations:** ^1^ Research Group of Sustainable Energy, Air and Water Technology Department of Bioscience Engineering University of Antwerp Groenenborgerlaan 171 Antwerpen 2020 Belgium; ^2^ Department of Biotechnology and Food Science Norwegian University of Science and Technology Trondheim 7491 Norway

## Abstract

The increasing world population and living standards urgently necessitate the transition towards a sustainable food system. One solution is microbial protein, i.e. using microbial biomass as alternative protein source for human nutrition, particularly based on renewable electron and carbon sources that do not require arable land. Upcoming green electrification and carbon capture initiatives enable this, yielding new routes to H2, CO2 and CO2‐derived compounds like methane, methanol, formic‐ and acetic acid. Aerobic hydrogenotrophs, methylotrophs, acetotrophs and microalgae are the usual suspects for nutritious and palatable biomass production on these compounds. Interestingly, these compounds are largely un(der)explored for purple non‐sulfur bacteria, even though these microbes may be suitable for growing aerobically and phototrophically on these substrates. Currently, selecting the best strains, metabolisms and cultivation conditions for nutritious and palatable microbial food mainly starts from empirical growth experiments, and mostly does not stretch beyond bulk protein. We propose a more target‐driven and efficient approach starting from the genome‐embedded potential to tuning towards, for instance, essential amino‐ and fatty acids, vitamins, taste,... Genome‐scale metabolic models combined with flux balance analysis will facilitate this, narrowing down experimental variations and enabling to get the most out of the ‘best’ combinations of strain and electron and carbon sources.

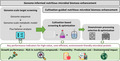

## Renewable H_2_‐ and CO_2_‐derived compounds as novel resource framework for microbial biomass production

Food production and the planetary boundaries are already beyond the limits of sustainability (Steffen *et al*., 2015), yet even more pressure on Earth’s carrying capacity is expected with the global population projected to reach 9.2–12.3 billion people by the turn of the century and the rise in living standards (Gerland *et al*., 2014). Structural changes of the agricultural‐based food chain are required to increase food production and simultaneously reduce the environmental footprint (Horton, 2017). An alternative route to conventional food is the production of microorganisms as a source of human food also known as microbial protein or single‐cell protein (Pikaar *et al*., 2018). The cultivation of microorganisms for food has many environmental benefits compared to agricultural crop production such as a reduction in arable land expansion, greenhouse gas emissions, nitrogen pollution and water use (Matassa *et al*., 2016; Pikaar *et al*., 2018).

Despite the environmental advantages of microbial protein as a food ingredient, studied and industrial production ways are mostly based on agricultural products or fossil fuels as a source of electron donors and/or carbon sources such as molasses, sucrose, starch, methane from natural gas, n‐alkanes and methanol (Nasseri *et al*., 2011). To truly revolutionize sustainable food production, uncoupling from agriculture or non‐renewable fossil fuels is needed, and hence another resource usage framework. Strong potential lies in the ‘green’ electrification of the chemical industry. This entails electricity production from photovoltaics or wind turbines during off‐peak hours followed by water or carbon dioxide (CO_2_) reduction with the renewable electron (Martens *et al*., 2017). First, hydrogen gas (H_2_) is generated through water electrolysis as a fuel for heat, energy or transportation in the so‐called hydrogen economy (Marbán and Valdés‐Solís, 2007). Secondly, CO_2_ is reduced into simple C1 or C2 building blocks such as methane (CH_4_), methanol (CH_3_OH), formic acid (HCOOH) and acetic acid (CH_3_COOH) as starting point for (bio)chemical synthesis a.k.a carbon capture and utilization (Martens *et al*., 2017; Satanowski and Bar‐Even, 2020). Renewable H_2_ or CO_2_‐derived compounds can then be used as an electron donor and/or carbon source for more sustainable microbial protein production (Pikaar *et al*., 2018; Linder, 2019).

**Fig. 1:**
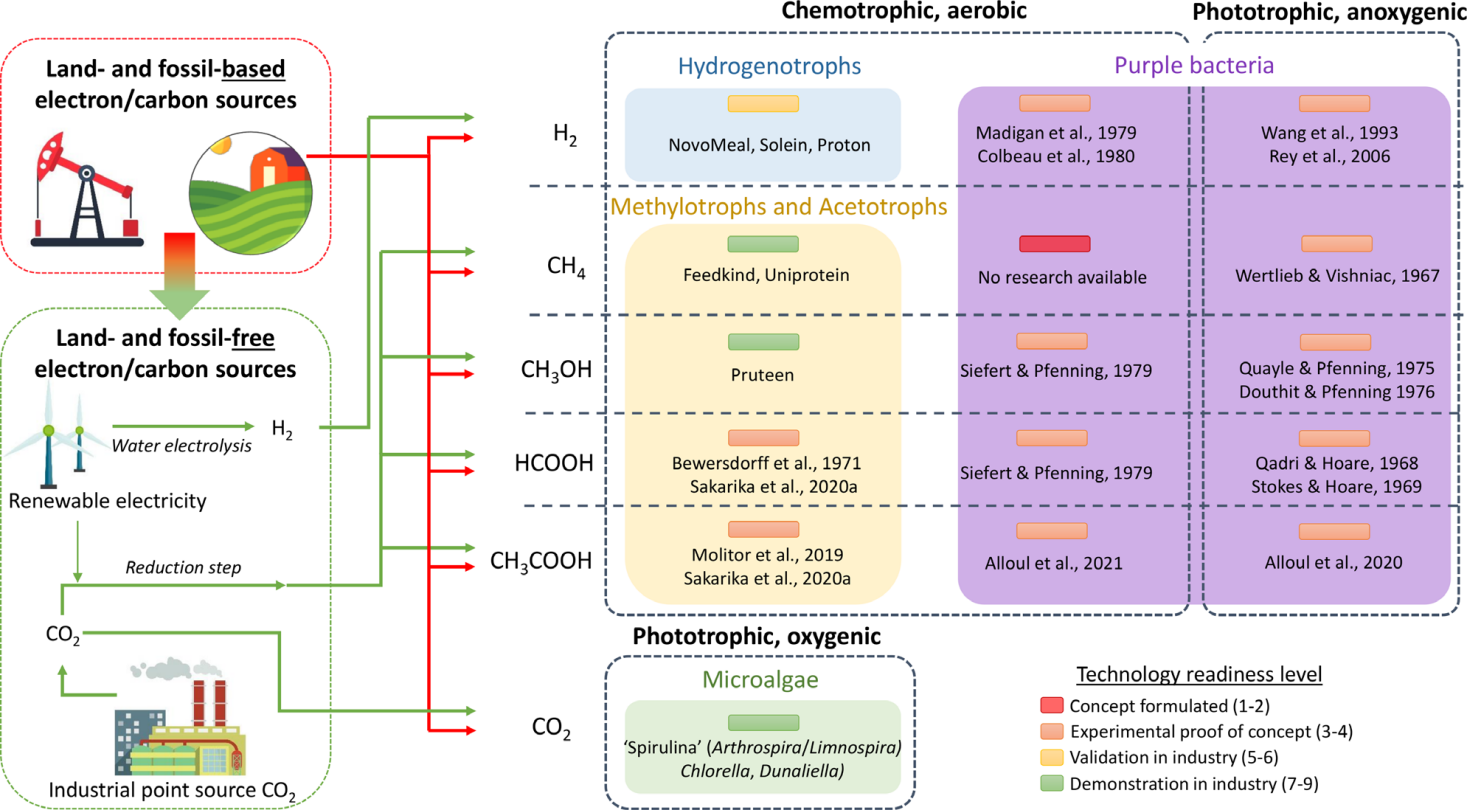
Transition towards electron donors and carbon sources that are not relying on arable land or fossil fuels, enabled by ‘green’ electrification of the chemical industry based on water electrolysis and carbon capture and utilization approaches. These routes can generate several electron donors and/or carbon sources such as hydrogen gas (H_2_), methane (CH_4_), methanol (CH_3_OH), formic acid (HCOOH), acetic acid (CH_3_COOH) and carbon dioxide (CO_2_), which are directed to the production of several major classes of chemotrophic and phototrophic microbes and metabolisms. A simplified technology readiness level (TRL), based on the Horizon 2020 work programme definitions, indicates the highest level of maturity of the microbial protein production technology. For commercial products (TRL 5‐9), examples of brand names are indicated, and for others, indicative scientific literature sources are given (TRL 1‐4). Copyright‐free images were sourced from Freepik.com.

The more traditional R&D targets for growing microbial food in a largely land‐ and fossil‐free manner are (aerobic) hydrogen oxidizing bacteria, methylotrophs, acetotrophs or (phototrophic) microalgae respectively cultivated on H_2_, C1 compounds, CH_3_COOH and CO_2_ (Linder, 2019; Fig. 1). Other potentially appealing microbes for microbial production are purple non‐sulphur bacteria (PNSB; Alloul *et al*., 2021b). They are typically explored photoheterotrophically for resource recovery and environmental technology (Alloul *et al*., 2018; Cerruti *et al*., 2020). Even though PNSB are extremely metabolic versatile, research studying their growth for aerobic or phototrophic hydrogen or methylotrophy is rather limited (Fig. 1).

Biomass cultivation for microbial protein in general, mainly aims at enhancing the growth rate and (bulk) protein content, rarely targeting nutritious compounds such as essential amino and fatty acids, vitamins and antioxidants or palatability such as taste, odour, texture and appearance. High nutritious biomass quality is, nonetheless, essential to better match the dietary requirements of humans and increase the monetary value of the product. Sensory experience or palatability is also key for the acceptance of food (Lawless, 1991). A study, for instance, showed no clinical side effects from microbial protein intake (12–15% of the daily required nitrogen), yet the taste was critical for the acceptance of food by the participants (Abrahamsson *et al*., 1971).

Currently, improving the nutritional biomass quality, palatability and growth rate is generally achieved by first selecting or modifying (ethical/legal challenging) the best strain and metabolism followed by optimizing the cultivation conditions (i.e. nutritional quality steering) to get nutrition‐wise the ‘best’ out of the genome. This is typically realized through a non‐targeted and iterative cultivation‐based methodology which is labour‐intensive and empirical. There is a lot of genomic data and techniques available to mine the potential of microbes, yet it is still not commonly applied in the field of microbial protein production. A new approach is, therefore, needed to unlock the full genome‐embedded potential of microbes for nutritious and palatable microbial food and enable more targeted and efficient experimenting.

## The usual microbial protein suspects on H_2_, CO_2_ and/or C1‐C2 compounds

To date, a variety of (metabolic groups of) microbes has typically been considered for microbial protein production on H_2_ and C1–C2 compounds with different technology readiness levels Fig. 1. The following section illustrates that strain selection has been briefly touched upon, yet optimizing the cultivation conditions and improving the palatability is often overlooked in literature and, if performed, based on a cultivation‐based approach.

Aerobic hydrogenotrophs, a.k.a. hydrogen oxidizing bacteria, are considered by several companies such as Deep Branch Technology (‘Proton’), NovoNutrients (‘NovoMeal’) and Solar foods (‘Solein’), which all state the importance for the transition towards renewable H_2_ for protein production (Deep Branch Biotechnology; NovoNutrients; Solar Foods). Unfortunately, none of these companies disclose the used species but *Cupriavidus necator* has predominantly been studied (Pander *et al*., 2020). Volova and Barashkov (2010) performed strain selection based on the essential amino acid profile for three species, which included *Cupriavidus necator* (formerly known as *Ralstonia eutropha*). However, most studies are focused on the optimization of polyhydroxyalkanoate production by aerobic hydrogenotrophs for valorization as bioplastics instead of maximizing nutritional compounds (Pander *et al*., 2020).

Production of Methanotrophs with CH_4_ as electron source, is currently the most mature technology on the market. Companies such as Unibio A/S (‘UniProtein’) and Calysta (‘Feedkind’) are using a strain of *Methylococcus capsulatus* (Calysta; Unibio Group). A study on strain selection has been performed by D'Mello (1972). They reported that, from six species, *M*. *capsulatus* had the highest crude protein content and *Methylomonas agile* showed to have the highest methionine and cysteine concentration. Literature on nutritional quality steering is more difficult to find and, probably, restricted to undisclosed company reports.

For CH_3_OH, a fully demonstrated technology was developed in the 1970s by Imperial Chemical Industries which used *Methylophilus methylotrophuson* (‘Pruteen’; Westlake, 1986). Abou‐Zeid and Baghlaf (1983) reported strain selection based on the essential amino acid profile for three species.

Aerobic microbial protein production on HCOOH and CH_3_COOH is still in the laboratory phase (Bewersdorff and Dostalek, 1971; Molitor *et al*., 2019; Sakarika *et al*., 2020a). While for both compounds strain selection has been performed by Sakarika *et al*. (2020a), no reports have been found, thus far, on nutritional quality steering. For palatability, on the other hand, HCOOH and CH_3_COOH are probably one of the few compounds that have been studied. A recent publication by Sakarika *et al*. (2020b) on HCOOH or CH_3_COOH showed that the type of culture (pure vs. mixed), species and compound affects the sensory properties.

Phototrophic microalgae production on CO_2_ has been extensively considered and developed for human and animal consumption, albeit more as functional food than as protein source, with numerous companies in Asia and North America (Ritala *et al*., 2017; Koyande *et al*., 2019). Screening of commercially available ‘Spirulina’ (genera *Arthrospira/Limnospira)* and *Chlorella* revealed a relatively large heterogeneity, and hence optimization potential (Muys *et al*., 2019). Microalgae are probably the most extensively studied microbes for strain selection and cultivation‐based nutritional quality steering. Most studies focus on improving the essential amino acid profile by species selection (Hempel *et al*., 2012; Muys *et al*., 2019) or by changing environmental factors (Ogbonda *et al*., 2007; Sui *et al*., 2019b). Essential fatty acids (Hempel *et al*., 2012), vitamins (Watanabe *et al*., 2002) and antioxidants (Richmond, 2008; Sui *et al*., 2019a) have also been explored through species selection and parameter optimization. In terms of palatability, microalgae are also thoroughly explored for flavour, taste and texture (Lafarga, 2019). Many commercial products are available in the form of capsules, powder or integrated in food (e.g. chocolate, crackers, pasta, etc.; (Lafarga, 2019).

## Purple bacteria for hydrogenotrophy, methylotrophy and acetotrophy?

PNSB are appealing microbes for nutritious biomass production. Their potential is, first, derived from their highly versatile metabolism (Imhoff, 2006; Alloul *et al*., 2020), which allows examining a variety of electron and energy sources to steer towards nutritious biomass. Secondly, they have an appealing intrinsic nutritious biomass composition rich in protein with a considerable amount of vitamins (e.g. vitamin B2, B6, C, E, D and folic acid) and carotenoid pigments (e.g. spirilloxanthin, rhodopin, okenone and rhodopinal; Sasaki *et al*., 1998).

Strain selection and cultivation‐based nutritional quality steering on land‐ and fossil bound electron donors and complex mixtures (e.g. waste streams) have been successful for PNSB. For example, photosynthetic pigments (antioxidant properties) are normally not induced under aerobic dark conditions, yet researchers found that cultivating PNSB at dissolved oxygen concentrations lower than 0.4 mg O_2_ l^−1^ triggers the pigment synthesis (Ghosh *et al*., 1994; Alloul *et al*., 2021a). Moreover, in previous research, we selected *Rhodobacter capsulatus* as the most promising PNSB based on growth rate (Alloul *et al*., 2019). In the same article, we also showed that mixtures of volatile fatty acids improve the growth performance of several pure and mixed PNSB cultures relative to individual volatile fatty acids. In another paper, we observed that fructose as a carbon source enhanced the protein content of several PNSB species compared to growth on volatile fatty acids, alcohols or other sugars (Alloul *et al*., 2021a).

Microbial protein production with PNSB on H_2_‐ and CO_2_‐derived compounds is largely unexplored and research is mainly limited to phenotypic screenings rather than strain selection or directed towards nutritional compounds. For example, for H_2_, only two articles were published exploring the fundamentals of chemoautotrophic growth of two *Rb. capsulatus* strains (Madigan and Gest, 1979; Colbeau *et al*., 1980). Photoautotrophic growth has also been studied for *Rb*. *sphaeroides*, *Rhodospirillum rubrum* and *Rhodopseudomonas palustris,* with a dedicated focus on the metabolism, genetic regulation and growth kinetics (Wang *et al*., 1993; Rey *et al*., 2006). For CH_4_, only one article from the 1960s claims that *Rps*. *gelatinosa* is able to incorporate CH_4_ into biomass (Wertlieb and Vishniac, 1967), yet no other research is available whatsoever to support this. CH_3_OH is better studied with one article from the 1970s performing a kind of strain selection based on phototrophic growth rates of 39 isolates. They showed that a particular strain of *Rhodoblastus acidophilus*, formerly known as *Rhodopseudomonas acidophila*, was most suitable for growth on CH_3_OH (maximum specific growth rate 2.3 d^−1^; Douthit and Pfennig, 1976). The chemotrophic growth, on the other hand, was not well characterized (Quayle and Pfennig, 1975). For HCOOH, Stokes and Hoare (1969) explored its assimilation on a metabolic level. Growth rates and yields were also studied (Qadri and Hoare, 1968; Siefert and Pfennig, 1979), yet no nutritious compounds nor nutritional quality steering were reported.

Overall, there are still several research opportunities and gaps related to aerobic or phototrophic hydrogen or methylotrophic PNSB production. More research is required for strain selection and cultivation optimization for the production of nutritious and palatable microbial food.

## Genome‐informed selection for nutritious biomass

**Fig. 2:**
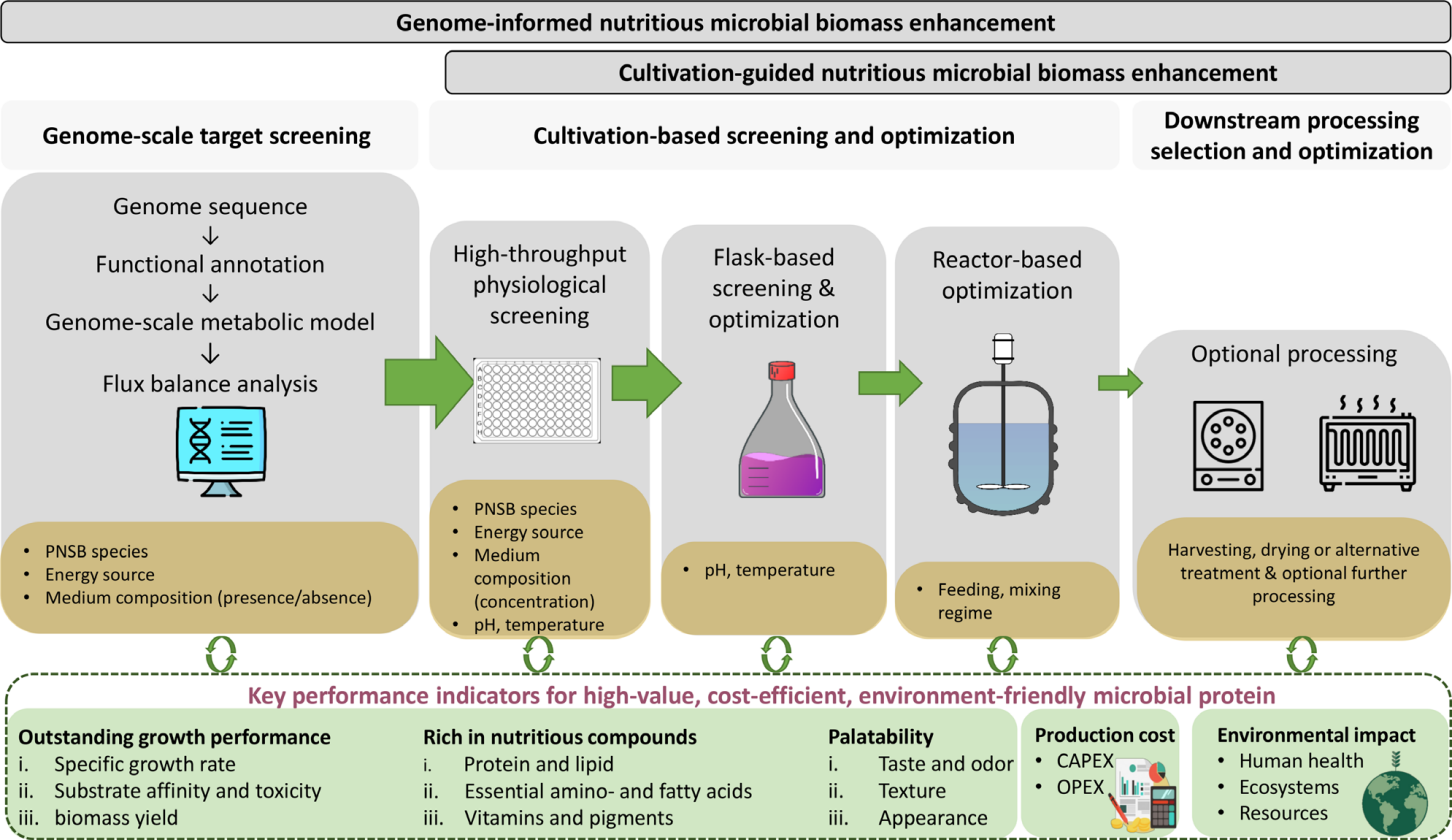
Proposed genome‐scale computational approach for targeted screening and nutritional quality steering. Grey boxes show the necessary steps in the route from computational prediction to downstream processing. Yellow boxes represent the variables of each step in the pipeline. The decreasing height of the green arrows depicts the number of variables (parameter values or options) to select in each step. Each step interacts with the key performance indicator. CAPEX, capital expenditure; OPEX, operational expenditure. This figure has been designed using resources from Freepik.com and flaticon.com.

Despite successful accomplishments, research aimed at enhancing the growth performance and nutritional quality of microbial biomass production remains labour‐intensive and is mainly empirical (Fig. 2). Investigations rarely start off with mining the genomic information, even though this is increasingly becoming available. The *nec‐plus‐ultra* approach to capitalize on the intrinsic biological potential of strains is the utilization of genome‐scale metabolic models (GEM; Oberhardt *et al*., 2009). These models account for every known metabolic reaction encoded in the genome of an organism and they can be generated from a genome assembly using automated metabolic reconstruction tools (Henry *et al*., 2010; Machado *et al*., 2018). GEM can be used to predict the metabolic phenotype of an organism under given growth conditions using flux balance analysis (FBA), a simulation method that calculates the optimal flow of metabolites through the metabolic network and its response to environmental and genetic perturbation. The combination of GEM and FBA allows to predict how the metabolic phenotype responds to different environmental and genetic perturbations and has become a popular computational framework for multiple biotechnological applications (Oberhardt *et al*., 2009).

In the context of nutritious microbial biomass production, this approach enables to mechanistically understand the effect of bioprocess variables such as electron donor and energy source on the relative changes in the fluxes of key pathways, such as those involved in the synthesis of essential amino and fatty acids, vitamins and pigments. This methodology will, therefore, allow narrowing down experimental variations and enable to get most out of the ‘best’ combinations of strain and electron and carbon sources (Fig. 2). Golomysova *et al*. (2010) for example, have used this approach on the *Rb*. *sphaeroides* for H_2_ production. From several individual and combined carbon sources, they accurately predicted that lactate resulted in the highest H_2_ productivity. A more extensive screening by Imam *et al*. (2011) tested the effect of different carbon, nitrogen and growth modes (e.g. aerobic and photoheterotrophic) on the growth rate, hydrogen and polyhydroxybutyrate production. *In silico* analysis corresponded well with the experimental observations and showed that H_2_ production was highest for a succinate glutamate mixture.

Although genome‐scale target screening is a powerful tool to predict relative changes in the fluxes of target pathways related to nutritional compounds, its main limitation lies in the fact that it falls short to compute the relative abundance of nutritional compounds in the biomass. In fact, the reconstruction of GEM requires a biomass objective function that describes the biomass composition of the organism in terms of the steady‐state concentration (mmol per gram of dry weight) of its individual components (amino acids, lipids, nucleotides, vitamins, cofactors). An accurately determined biomass objective function is essential to correctly estimate the growth rate and biomass yield on different compounds, yet for the sake of automation, this information is usually extracted from phylogenetically‐close model organisms. Currently, one can only infer how the abundance of a given compound is affected through different perturbations by observing the response at the level of its respective pathway fluxes.

Recently, a variety of methods have been proposed to replace the biomass objective function with objective functions based on gene expression data, but their predictive power seems limited (Machado and Herrgard, 2014). Lachance *et al*. (2019) propose a computational workflow to generate a species‐specific biomass objective function, but it still requires experimental determination of the relative fraction of the main macromolecules (DNA, RNA, proteins and lipids), and gene expression data to determine the abundance of individual nucleotides and amino acids (Lachance *et al*., 2019). A new class of models of metabolism and gene expression (ME‐models) extends the traditional GEM by explicitly modelling all the biosynthetic machinery (ribosomes, nucleotides and proteins; O'Brien *et al*., 2015). ME‐models can fully predict gene and protein expression as well as the biomass composition. However, their reconstruction requires a large knowledgebase and a significant amount of manual curation, making them still unsuitable for integration into a fast‐screening process.

In summary, genome‐driven enhancement of microbial food development shows great potential, particularly based on GEM. The combination of GEM and FBA is the most established and applicable approach as a promising alternative to the typical empirical cultivation‐based methodology. As researchers did successfully for other examples of GEM applications, it is time to embark on showing the efficacy of such approach in selecting the best combinations of strain, metabolism and cultivation conditions. This approach may facilitate the ultimate goal of industrially producing very nutritious and palatable microbial food in a cost‐effective and ecological manner.

## Acknowledgements

The authors would like to kindly acknowledge the DOCPRO4 project ‘PurpleTech’ funded by the BOF (Bijzonder onderzoeksfonds; Special research fund) from the University of Antwerp, and the project 'Saraswati 2.0' (821427) funded by the European Union’s Horizon 2020 Research and Innovation programme, for financial support of J.S. and A.A., respectively.
